# The effects of hearing protection devices on spatial awareness in complex listening environments

**DOI:** 10.1371/journal.pone.0280240

**Published:** 2023-01-12

**Authors:** Hillary A. Snapp, Barbara Millet, Natasha Schaefer-Solle, Suhrud M. Rajguru, Sebastian A. Ausili

**Affiliations:** 1 Department of Otolaryngology, University of Miami, Miami, FL, United States of America; 2 Department of Interactive Media, University of Miami, Miami, FL, United States of America; 3 Department of Medicine, University of Miami, Miami, FL, United States of America; 4 Department of Biomedical Engineering, University of Miami, Miami, FL, United States of America; Military Institute of Science and Technology, BANGLADESH

## Abstract

Hearing protection devices (HPDs) remain the first line of defense against hazardous noise exposure and noise-induced hearing loss (NIHL). Despite the increased awareness of NIHL as a major occupational health hazard, implementation of effective hearing protection interventions remains challenging in at-risk occupational groups including those in public safety that provide fire, emergency medical, or law enforcement services. A reduction of situational awareness has been reported as a primary barrier to including HPDs as routine personal protective equipment. This study examined the effects of hearing protection and simulated NIHL on spatial awareness in ten normal hearing subjects. In a sound-attenuating booth and using a head-orientation tracker, speech intelligibility and localization accuracy were collected from these subjects under multiple listening conditions. Results demonstrate that the use of HPDs disrupts spatial hearing as expected, specifically localization performance and monitoring of speech signals. There was a significant interaction between hemifield and signal-to-noise ratio (SNR), with speech intelligibility significantly affected when signals were presented from behind at reduced SNR. Results also suggest greater spatial hearing disruption using over-the-ear HPDs when compared to the removal of high frequency cues typically associated with NIHL through low-pass filtering. These results are consistent with reduced situational awareness as a self-reported barrier to routine HPD use, and was evidenced in our study by decreased ability to make accurate decisions about source location in a controlled dual-task localization experiment.

## I. Introduction

Noise-induced hearing loss (NIHL) is the second most common cause of hearing loss after age-related hearing loss [1], with noise exposure now recognized globally as the leading cause of acquired hearing loss in adults. In fact, 24% of hearing difficulty among U.S. workers is caused by occupational exposures making hearing loss the most common occupational impairment in the U.S. [2]. Despite increasing awareness of NIHL as a major occupational health problem, implementation of effective hearing protection interventions remains challenging [3–5]. Reduction of situational awareness has been reported as a primary barrier to hearing protection device (HPD) use in at-risk populations such as the fire service and military [3, 6–10].

Accurate sound localization contributes to situational awareness and influences rapid decision making. HPDs both reduce overall access to sound and disrupt access to auditory cues responsible for key spatial orientation tasks such as localization accuracy and front-back distinction [11–15]. Occupational NIHL typically reduces access to high-frequency spectral cues, which may serve to further disrupt localization abilities [16–19]. Disruption of these cues may not only compromise localization, but also disorient the listener and affect reaction times. The ability to quickly locate and orient to targets of interest is essential to safety and job performance for several emergency response workers (ERWs) focused on public safety such as police officers, correction officers, firefighters, and emergency medical response workers who regularly respond to life-threatening calls to action under stressful and hazardous conditions.

Reduced situational awareness has been identified as one of the major causes of workplace injuries and fatalities [20–25]. Moreover, the importance of spatial perception for worker safety has been emphasized by the Occupational Safety and Health Administration for several occupations [26–29]. Precise and timely analysis of the environment is critical in emergency response occupations. ERWs work in challenging, hazardous situations and must manage multiple tasks simultaneously. This is further complicated by the need to detect and identify the location of a sound source, most often with interfering signals which create poor or even negative signal-to-noise ratios (SNRs). For this reason, HPD use continues to be perceived by workers as increasing risk for operational injuries and fatalities [10, 30].

A large body of research points to the importance of auditory localization for situational awareness and orientation, and previous studies have shed light on potential factors which may influence localization abilities in emergency response situations. This includes findings that stimuli are better localized when they are broadband [31, 32], longer in length [33], and salient [34, 35]. While prior studies have explored the impact of HPDs and NIHL on spatial perception, an important limitation is the common use of impulse or short duration stimuli [15, 36–43], which lacks ecological validity for many non-combat related emergency response situations. Public Safety operations often entail communication tasks that require the listener to monitor, process, and respond to two or more simultaneous speech streams [44]. Continuous monitoring of multiple channels of communication is an essential duty of ERWs, making performance in multi-talker environments of critical importance for those working in public safety. For example, listeners will employ active head movement to make use of the interaural difference cues required for localizing an auditory event in space [45, 46]. Competing noise is known to negatively affect localization accuracy [47]. In multi-talker environments, uncertainty of the target signal [48], would be expected to affect localization accuracy. At the same time, there is an expected benefit of the spatial separation of the target from the masker. In competing noise, listeners may re-orient themselves to improve the SNR, and in turn, enhance their performance [49, 50]. However, re-orienting as a strategy is typically ineffective for short duration stimuli [50, 51]. Moreover, studies show that the greatest effect of spectral cue disruption on localization is at +/- 90 degrees azimuth and that localization is minimally affected by NIHL for targets directly in front of the listener [16]. Re-orienting to the target may require greater effort, but may also allow for improved accuracy. Further, there is little information on the effect of other competing tasks on auditory localization, inherent in essential duties of ERWs.

We are interested in studying behaviors that listeners may employ to improve their performance under challenging listening scenarios where they may be required to perform more than one task simultaneously. The objective of the current study was to investigate the effect of HPDs and NIHL on spatial perception in a dual-task experiment. Here, we use both spatial and speech paradigms to investigate localization abilities across task modality. In addition to HPDs, we employed low-pass filtering to simulate NIHL in normal hearing listeners to allow for within-subject comparison of how limited frequency bandwidth would affect localization, speech intelligibility, and reaction times.

## II. Methods

This research was conducted according to the principles expressed in the Declaration of Helsinki and was approved by the Institutional Review Board at the University of Miami (IRB#20210223).

### A. Participants

Ten normal hearing adults participated in this study. The participants ranged in age from 20 to 46 years (M = 35.8, SD = 6.9). There were 2 males and 8 females. All participants had normal hearing bilaterally, as determined by air conduction hearing thresholds of < 20 dB HL across the independent standard audiometric test frequencies, 250–8000 Hz, and no prior history of hearing impairment. Written consent was obtained for all participants.

### B. Experimental setup

**Fig 1:**
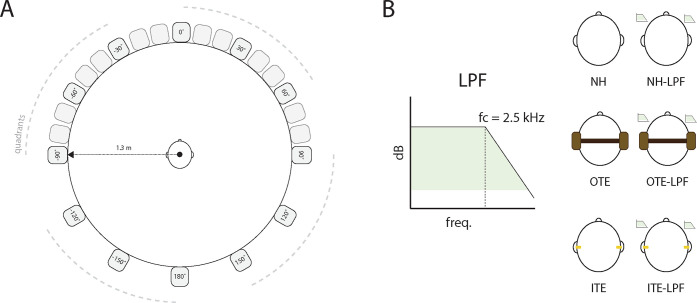
Illustration of the experimental setup and listening conditions. A) Participants were seated in the center of a 24-speaker array. The 12 speakers used to present the stimuli are indicated by their location in azimuth at a speaker-to-speaker distance of 30°. A single talker was presented from each quadrant for every trial, indicated by the grey dotted lines. B) The 6 listening conditions (normal hearing, with low-pass filtering, and with over-the-ear (OTE) or in-the-ear (ITE) HPDs, see section E) and the low-pass filtering applied to simulate NIHL are visualized.

The experiment took place in a sound attenuated auditory booth (4.3 x 4.3 x 2m). During the task, listeners sat in the center of a 24-speaker circular array (Mix Cube, Avantone, NY, USA) spanning 360° with a radius of 1.3m (Fig 1). Sound presentation was driven by a 24-channel sound card (MOTU 24Ao, Cambridge, MA, USA), connected to three amplifiers of eight channels each (Crown CT 875, Los Angeles, CA, USA). Stimuli presentation and the analysis of the responses were implemented using custom script written in MATLAB (ver. R2020b, The MathWorks, Natick, USA), the Psychophysics Toolbox extension, and the Lab Streaming Layer library for device time synchronization (https://github.com/sccn/labstreaminglayer). A lightweight, non-intrusive, custom head tracker with laser pointer and 9-axis inertial measurement unit fixed to a three-dimensional printed spectacle frame was used to obtain head rotations in response to stimuli.

### C. Stimuli

Stimuli consisted of target sentences from the coordinated response measure (CRM) corpus (36). All CRM sentences have the same structure: “ready (call-sign) go to (color) (number) now”, and consist of eight possible call signs (Arrow, Baron, Charlie, Eagle, Hopper, Laker, Ringo, Tiger), four colors (blue, red, green, white), and eight numbers (1–8). These combinations yield 256 different sentences recorded for eight different talkers giving a total of 2048 sentences in the corpus. The CRM stimuli are broadband in nature and embedded in multiple competing speech streams, allowing for the investigation of how listeners may leverage available spatial perception cues in complex environments while managing more than one task simultaneously (i.e., target identification, target localization, and speech intelligibility).

### D. Procedures

Four speech streams, randomly selected from the CRM corpus [36], were presented simultaneously to the listener through four speakers in a 360° horizontal array, with each quadrant represented in every trial (Fig 1A). Twelve of the 24 visible speakers separated by 30° were used to present the stimuli (Fig 1A). Participants were not made aware of which speakers the stimuli were presented from or the number of speakers used in the experiment.

Listeners were instructed to listen for the target talker, which was identified by the presence of the call sign “Hopper” amidst three different interfering talkers. All talkers were male. Target stimuli were randomly presented at +5 and +10 SNR with the interfering talkers presented at 70dBA. Pilot data collected before the primary experiment indicated these SNRs appropriately minimized performance ceiling and floor effects. The stimulus and competing speech levels were verified at the ear level of the listener with signals calibrated individually to ensure the designated SNR as measured at the location of the listener. The sentences in the speech corpus were synchronized at their onsets (“Ready”) [52].

The experimental task required the listener to 1) locate the source of the target talker in azimuth, and 2) report (identify and recall) the associated color/number (e.g., “Blue” “One”). During the task, participants sat comfortably in a chair and were instructed to point a head-mounted LED towards the perceived sound location via head movement. Head orientation and velocity of the head movement were recorded during sound presentation and analyzed offline to obtain localization accuracy. Prior to each trial, subjects had to fixate at 0° azimuth directly in front of the participant to ensure proper head orientation (calibrated using the head tracker).

### E. Listening conditions

Listeners were tested using both over-the-ear (OTE) and in-the-ear (ITE) HPDs. E-A-Rsoft™ FX™ foam earplugs (3M™ New Zealand Pty, Limited), with a Noise Reduction Rating (NRR) of 33 dB were used for the ITE listening conditions. At the tolerance of the participant, deep insertion of the plug was obtained by ensuring the outside edge of the earplug was flush with the entrance of the ear canal [53]. PELTOR™ Optime™ 105 Earmuffs (3M™ New Zealand Pty, Limited), with an NRR of 30 dB were used for the OTE listening conditions.

Since individuals with NIHL tend to experience hearing loss at 3000 Hz and above, the signal was low-pass filtered using a cutoff frequency of 2.5 kHz with an order of 100 so that the audibility of the filtered speech was representative of individuals with impaired high-frequency hearing arising from noise exposure. The experiment was conducted with six listening conditions using a within-subjects design (shown in Fig 1B): 1) normal-hearing (NH), 2) NH with low-pass filter (NH-LPF), 3) OTE, 4) OTE with LPF (OTE-LPF), 5) ITE, and 6) ITE with LPF (ITE-LPF). Each listening condition consisted of 96 trials for a total of 576 trials, with 288 presented at +5 dB SNR and 288 presented at +10 dB SNR randomly in an interleaved manner. All listeners started in the NH listening conditions. HPD listening conditions were counterbalanced (i.e., half the participants began with the OTE listening conditions and half with the ITE listening conditions). The LPF conditions were always anchored to their associated hearing condition (i.e., OTE to OTE-LPF) so as not to introduce intra-subject variability by disrupting the positioning of the HPD and thus attenuation at the ear.

### F. Data analysis

Speech intelligibility was calculated as the percentage of color and number stated by the target talker that were correctly repeated by the participant. For analysis, percent correct scores were converted to rationalized arcsine units (RAU) [54]. The rationalized arcsine transform produces a scale with units that have almost the same size as percentages while satisfying the assumptions of statistical procedures that are used to analyze the scores. The arcsine transform is used to express percent correct scores in radians (Eq 1) and the rationalized arcsine transform adjusts the scores into units for analysis (Eq 2):

$$AU=\arcsin\sqrt{\frac{x}{N+1}}+\arcsin\sqrt{\frac{x+1}{N+1}}$$ (1)

$$RAU=\left(\frac{146}{\pi }\right)*AU-23$$ (2)

where X is the total number of colors and numbers that were correctly repeated by the participant and N is the number of trials performed.

**Fig 2:**
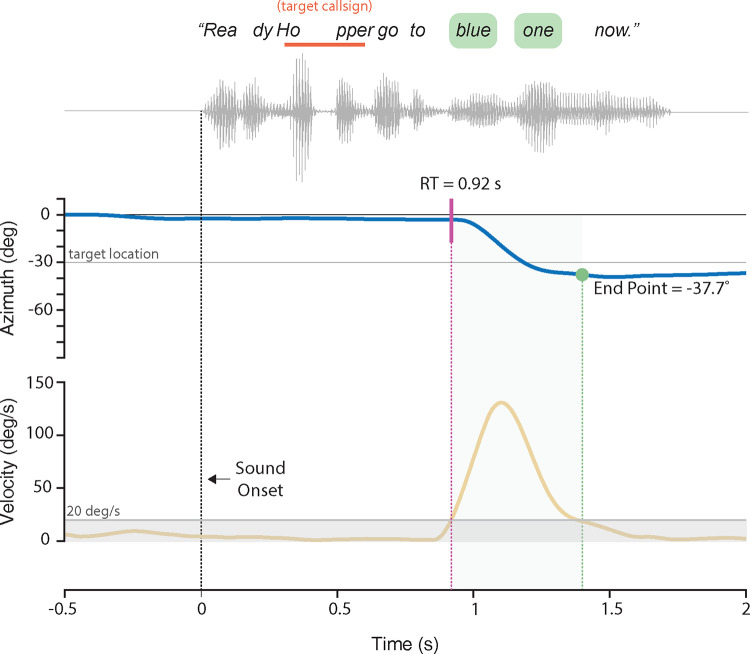
Analysis of localization response using head tracking. A sample target stimulus from the CRM corpus is presented with its associated waveform. The target call sign used was “hopper” and the associated color/number to be repeated are indicated in green. The upper panel shows the head position in azimuth (degrees) and lower panel shows the velocity of the head movement (degrees/second). RT is calculated as the time between the onset of the stimulus and initiation of the head movement. The endpoint of the head movement is set once its velocity profile falls below 20°/s (grey shaded bar).

Head-orienting responses were characterized by a saccadic profile (i.e., rapid, step-like movement of monophasic velocity). Head-orienting responses were automatically detected using a custom-made MATLAB script that identified head velocities exceeding 20°/s (Fig 2A). This analysis determines onset (vel. > 20°/s, start of head movement) and offset (vel. < 20°/s, when the head is static again). These markers were visually checked offline by the experimenter on a trial-by-trial basis. The perceived target’s speaker location, or end point, was determined by the offset head’s position (in degrees, Fig 2). The overall measure of the localization response accuracy was processed by computing the mean absolute error (MAE) across trials as follows:

$$MAE=\frac{1}{N}\sum_{n=1}^{N}\left|\alpha_{R}^{n}-\alpha_{T}^{n}\right|$$ (3)

where R is the response and T is the target (in degrees). The reaction time (RT) relative to the stimulus presentation is defined by the onset of the head rotation (in sec, Fig 2). For quantitative analysis, RT data were transformed to its reciprocal, known as the response promptness (in s^-1^), which is shown to follow a Gaussian distribution [55].

Dependent variables included speech intelligibility, localization, and response promptness. Results were analyzed using repeated measures analysis of variance (RM-ANOVA) with three within-subject factors (listening condition [NH, NH-LPF, OTE, OTE-LPF, ITE, ITE-LPF], SNR [+10 dB, +5 dB], and hemifield presentation [front, back]). A significance level of α = 0.05 was applied. Pairwise comparisons with Bonferroni corrections for multiple comparisons were used to follow-up significant main effects. Correlation analysis was also performed to evaluate the predictive value of the independent variables. All analyses were performed using SPSS® software (version 26.0; New York: IBM Corp®).

## III. Results

### A. Speech intelligibility

**Fig 3:**
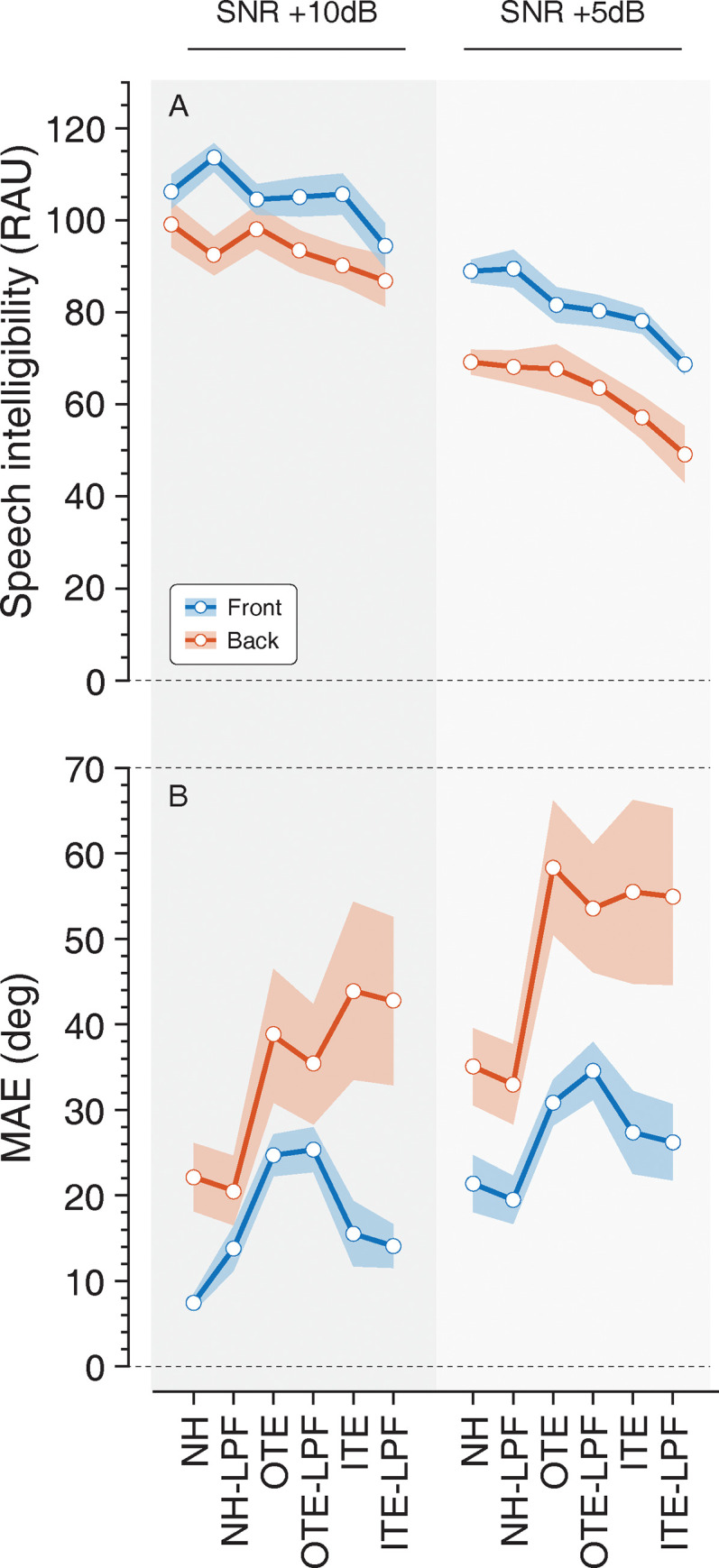
Listeners’ speech intelligibility performance is shown as overall percent correct identification of color and number spoken by the target talker, and localization performance is presented in MAE for all listening conditions. Here, lower MAE values indicate better localization performance. Performance for targets presented to the front is shown in blue and to the back in red. Shaded areas show standard error of the mean.

Listeners performance for the speech intelligibility task is presented in Fig 3A. Analyses revealed significant main effect of hemifield [*F*(1,9) = 22.86, *p* < .001, *η*_*p*_^2^ = .718], a significant main effect of SNR [F(1,9) = 424.19, p < .001, η_p_^2^ = .979], and a significant main effect of listening condition [F(5,45) = 6.99, p < .001, η_p_^2^ = .437] on listeners’ ability to correctly identify the color and number stated by the target talker. Specifically, there was a significant difference in performance when signals were presented from the front compared to rear presentation (Mean difference = 15.1 RAU, *p <* .*01*), and between +10 dB SNR and +5 dB SNR (Mean difference = 27.3 RAU, *p <* .*001*), with performance in the rear and at +5 dB SNR being significantly worse. Pairwise comparisons revealed a mean difference of 16.1 RAU between the NH condition and the ITE-LPF condition (p = .01). There was a significant interaction between hemifield and SNR [F(1,9) = 6.86, *p <* .*05*, η_p_^2^ = .437], suggesting that the effect of hemifield on speech intelligibility was greater at the reduced SNR, and that the effect of signals presented from behind on speech intelligibility was greater at the reduced SNR. There were no interactions of hemifield and listening condition (*p =* .*09*), SNR and listening condition (*p =* .*41*), or hemifield by SNR by listening condition (*p =* .*60*). Pairwise comparisons revealed a mean difference of 20.0 RAU between the NH condition and the ITE condition (p < .001) and 20.8 RAU between the NH condition and the ITE-LPF condition (p < .05) when signals were presented in the front at +5 dB SNR.

### B. Localization

Fig 3B presents the localization performance. Analyses revealed a significant main effect of hemifield [F(1,9) = 10.55, p = .01, η_p_^2^ = .54], a significant main effect of SNR [F(1,9) = 63.68, p < .001, η_p_^2^ = .876], and a significant main effect of listening condition [F(5,45) = 4.94, p < .05, η_p_^2^ = .354]. In general, listeners’ localization of the target talker was better (i.e., lower MAE) for signals presented to the front (hemifield Mean difference = 19.42°, *p = 0*.*01)* and at the more favorable +10 dB SNR (Mean difference = 12.17° *p <* .*001*). This result extends the existing literature on localization abilities where best performance occurs for stimuli located in the front hemifield [56, 57], and at higher SNRs [35, 47]. There was also a significant interaction between hemifield and listening condition [F(5,45) = 3.33, *p =* .*01*, η_p_^2^ = .270]. Pairwise comparisons revealed significant differences in localization abilities for signals presented to the front from the NH to OTE listening condition (*p =* .*001*), NH to OTE-LPF listening condition (*p <* .*05*), and NH-LPF to OTE listening condition (*p <* .*01*) at +10 dB SNR; NH to OTE-LPF listening condition (*p <* .*01*), NH-LPF to OTE listening condition (*p <* .*01*), and NH-LPF to OTE-LPF listening condition (*p <* .*05)* at + 5 dB SNR. No significant differences by listening condition were observed for signals presented from the rear. Furthermore, there were no significant interactions between hemifield and SNR (p = .227), SNR and listening condition (*p =* .*91*), or hemifield by SNR by listening condition (p = .41).

### C. Promptness

**Fig 4:**
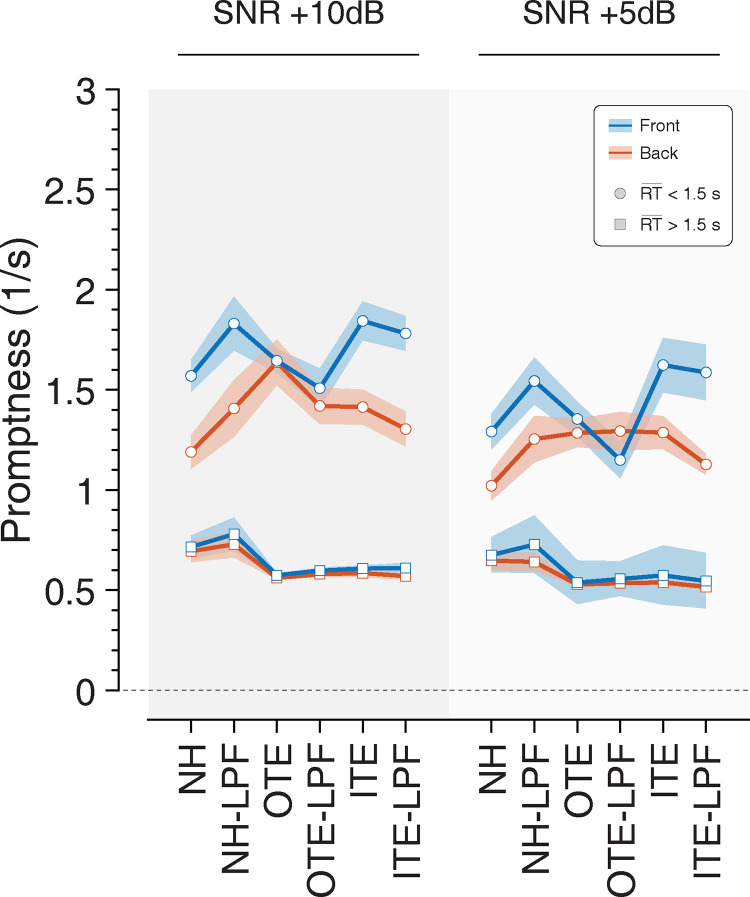
Mean response promptness (inverse of RT) across subjects for each listening condition, SNRs and font/back hemifields (blue/red, respectively). Shaded areas show standard error of the mean. Participants with measurable RTs are indicated by circles. Those participants whose average RTs across listening conditions exceeded a cutoff of 1.5 s are indicated by squares.

Response promptness (the reciprocal of RT) for SNR and listening condition is shown in Fig 4. Analyses revealed a significant main effect of SNR [F(1,9) = 26.389, p < .001, η_p_^2^ = .746] and hemifield [F(1,9) = 16.606, p < .01, η_p_^2^ = .649]. There was no significant effect of listening condition [F(5,5) = .808, p = .59, η_p_^2^ = 447]. RTs slowed when signals were presented from the rear compared to the front, and at +5 dB SNR compared to +10 dB SNR. There were no significant two-way interactions between hemifield and SNR (p = .121), hemifield and listening condition (p = .194), SNR and listening condition (p = .549), or three-way interaction among hemifield, SNR, and listening condition (p = .553).

We identified a subset of subjects (n = 3) that showed lower overall values (slower responses) than other respondents. The overall mean CRM sentence length was ~1.8 s. Closer review of the data revealed that these subjects waited until the color/number of the sentence was presented to then orient the head towards the perceived target location. This behavior was irrespective of condition, SNR, or hemifield. Fig 4 presents this difference in behavioral pattern for those subjects having an overall reaction time <1.5s (circles, Fig 4) and >1.5s (squares, Fig 4). As expected, there is no difference in promptness of the response for slow subjects for hemifield, SNR, or any conditions. In those who were fast, a significant difference in mean response was observed for hemifield (.25 s, *p <* .*01*) and SNR (.21 s *p <* .*001)*, but not for any of the listening conditions, consistent with the overall results.

### D. Influencing factors on performance

Although hemifield and SNR influenced speech intelligibility, Pearson correlation analysis revealed that localization accuracy did not influence speech intelligibility. There were also no significant correlations between the promptness of the response and localization, or promptness of the response and speech intelligibility. Combined, these results suggest that speech intelligibility is not influenced by how fast or accurate listeners are in identifying and locating the target. Yet, a closer review of the response patterns revealed different behavioral strategies between listeners.

**Fig 5:**
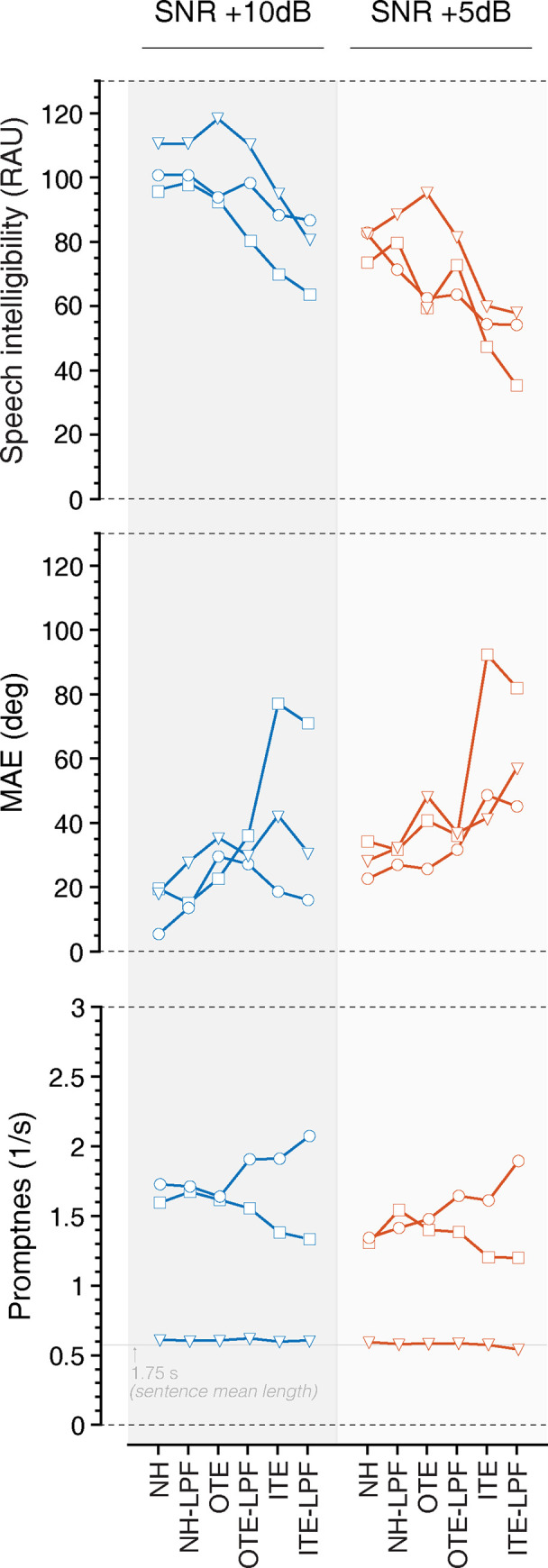
Representative examples of three listeners’ strategies highlight a tradeoff between speech intelligibility, localization, and response promptness. (A) accuracy for color and number identification, (B) MAE (localization performance), and (C) promptness (inverse of RT) are shown for participant 1 (O), 2 (□), and 3 (Δ). Behavior in +10 dB SNR is shown in the left column and +5 dB SNR in the right column of the figure.

Fig 5 shows a representative example of 3 listeners’ strategies for speech intelligibility, localization, and promptness. Interestingly, the listener whose promptness increased (see circles, Fig 5) as conditions became more challenging (i.e., lower RTs) also had the smallest localization error (MAE) and the best speech intelligibility (% Correct) in the +10 dB SNR condition. This does not hold true at +5 dB, suggesting that this strategy may not be effective in increasingly poorer SNRs. This effect has been observed in other domains where individuals who take longer to initiate a response outperform those who are quick to respond [58]. Longer processing times have been attributed to “information gathering”, which services to improve accuracy [59]. The second participant’s behavior (see squares, Fig 5) compromised speech monitoring and decreased promptness (increased reaction times), revealing decreased ability to manage dual tasks when compared to the other listeners and in the NH condition. The strategy of the third participant (see triangles, Fig 5) indicates a tradeoff between reaction time and accuracy, and prioritization of accuracy of the word task over localization.

## IV. Discussion

This study explored the impact of HPDs and NIHL on spatial perception. Results indicate that listeners are highly accurate at both localization and speech monitoring under the NH condition. Localization performance was negatively affected by HPDs, with significant effects observed in the OTE and OTE-LPF listening conditions when compared to the NH condition. Previous studies have demonstrated increased localization errors with loss of spectral cues either through the use of a low-pass filter [60–62], or through the use of HPDs [6, 11, 13, 14, 37, 39]. NIHL was simulated by removal of spectral information from the speech signals using a high order low-pass filter set at 2.5 KHz. While performance decreases under simulated NIHL conditions using low-pass filtered speech streams, the change is not significant for any measure and marginal compared to the changes observed with the OTE HPDs. This may be attributed to use of a longer speech stream compared to short noise bursts or single words [15, 36–43], which allows the listener to apply a search strategy to locate the target. Head movements are known to assist the listener in resolution of ambiguities in source location [50, 61–63]. The length of the CRM stimuli provided listeners with ample time to initiate head movements to disambiguate the spatial acoustic cues for localization of the target. Listeners are thereby able to employ inherent behavioral strategies to combat cue disruption arising from occlusion, loss of spectral cues, or competing sound sources. Investigating the role of different ecologically valid sources of interest provides new insights into the barriers of HPD use in emergency response operations, and how the disruption of spatial perception cues through HPDs or NIHL affects task performance. Specifically, impulse stimuli may not accurately reflect performance or spatial perception abilities when continuously monitoring multiple channels of communication.

This experiment employed a dual-task design where the listener had to actively locate the target signal and repeat the associated color and number from the target sentence. Target location was presented at random, unlike in previous studies where the target for speech-in-noise was always presented from a fixed and known location directly in front of the listener [42, 43]. The resulting cues available to the listener varied with each stimulus presentation, as the target could be presented anywhere in the 360° array. The ability to move the head during the stimuli presentation in the present experiment, likely allowed the listeners to leverage available interaural cues to facilitate locating the target and improve the SNR to optimize speech intelligibility.

As seen in Fig 3, the use of HPDs seems to be more disruptive than the low-pass filter alone (NH-LPF). There was no significant effect of filtering out high frequency spectral information from the speech stimuli on localization abilities in the azimuthal plane for any of the conditions. These findings suggest that the disruption caused by OTC HPDs may exceed that which arise from NIHL under certain scenarios, specifically at favorable SNRs, and/or for longer stimuli such as speech streams. Targets presented in elevation may have been more disrupted by the low-pass filtered speech, although head movement has been shown to reduce errors in elevation as well [64, 65]. Additionally, while overall MAE increases in the ITE HPD conditions, the change in MAE from the NH condition did not reach significance. Others have found that OTE HPDs are more disruptive to localization abilities than ITEs [13, 39, 66], and can be attributed to the additional loss of pinna cues which provide direction-dependent filtering for high frequency signals. Pinna cues are essential for localization in elevation [67], although, our findings present a clear affect in azimuth. Others have also demonstrated disruption of localization in the azimuthal plane when spectral cues are perturbed [68]. Here, even when the signal is longer in duration, allowing for the listener to re-orient to improve their performance, the ability to make accurate decisions about the location source in azimuth is negatively affected by HPDs. This affect is amplified as the competing signals create greater interference with the target (i.e., +5 dB SNR).

Results indicate that OTE HPDs are disruptive to localization abilities. This is consistent with that reported by ERWs [3, 6–8]. However, targets presented in the front hemifield at a favorable SNR are fairly well located when considering the complexity of the tasks. The MAE for signals presented to the front is < 30° (Fig 3), which may allow for sufficient performance on tasks such as search and rescue, particularly when integrated with other inputs (i.e., audiovisual integration). That is that the degree of error, although insufficient for the localization of impulse stimuli such as gunfire [43], is such that a listener could presumably locate the region or relative area of the source when presented in the front hemifield. Seemingly, a search to locate strategy could then be effective in quickly locating a target. The timing and effort required for signals presented from the rear, however, is perhaps too great for head orientation to facilitate localization of acoustic streams. Interestingly, there were no significant findings with ITE HPDs. This may be due to the small sample size in combination with the large variance in MAE across these conditions.

The ability of listeners to correctly identify and recall the associated color and number spoken by the talker using the call sign “Hopper” decreased when using HPDs, with significant decrease in performance for the ITE-LPF condition (Fig 3A). Speech intelligibility was also observed to be consistently better when the target originated in front of the talker compared to the rear. Larger differences in speech intelligibility were observed in the +5 dB SNR when compared to the +10 dB SNR across the various listening conditions, possibly pointing to the increase in cognitive demand that comes with making the SNR more difficult. These results indicate that disruption of spectral cues has a much greater effect on localization and speech intelligibility when the SNR is poor. Although the experiment was not designed to directly measure cognitive demand, it is possible that increased cognitive resources were required to perform the dual-task of localizing the target while also identifying and recalling the color and number stated by the talker. Here the target signal is both reduced in spectral detail while also masked by competing talkers, thereby requiring an increase in cognitive resources [69]. Collectively, this may also elucidate the findings observed in listeners on the localization and speech intelligibility task.

Changes in reaction times may represent disruption in situational awareness and have been suggested to reflect an increase in listening effort required for a given task. Bolia and McKinley showed increased reaction times for listeners under HPD conditions [36]. Smalt and colleagues showed that the use of HPDs increased listening effort, and suggested that HPD use led to cognitive fatigue in noisy environments [42]. In our cohort, promptness was highly variable across listeners, but as a group did not change significantly with HPD use.

Listeners performed a series of spatial and verbal tasks of increasing difficulty in a complex acoustic scene. Results demonstrate that HPDs impact spatial perception such that low SNRs were more susceptible to disruption of target identification and speech intelligibility relative to higher SNRs, with negative impacts to both target location accuracy and speech intelligibility. However, when the target signal was more salient and positioned in the front hemifield, listeners were better able to locate the talker and identify the talker content. There was no additive effect when the signal was low-pass filtered to simulate NIHL, suggesting that the negative effects HPDs on spatial perception may exceed the loss of high frequency cues associated with NIHL for monitoring of speech streams.

Because the sample used in this study consisted of normal hearing participants and used low-pass filtering to simulate NIHL, the findings from this study may not directly translate to ERWs. In addition, the design is not able to fully represent real world scenarios that ERWs may encounter. Future studies should include specific metrics to further explore the potential effects of NIHL and HPD use on cognitive resources.

## V. Conclusions

Use of HPDs can disrupt spatial perception in different ways, with the effect varying with the type of HPD. OTE HPDs significantly impacted localization performance, while ITE HPDs significantly impacted monitoring of speech signals. These results are consistent with the self-reported barrier to HPD use of reduced situational awareness and decreased task performance, evidenced by decreased ability to make accurate decisions about the location source in a controlled, dual-task localization experiment. Occlusion of the ear results in perceptible acoustic disruption of sound, and reduced access to spatial cues. The results further suggest that behavioral strategies are ineffective for improving performance in increasingly adverse listening scenarios. The disruption of spatial perception cues associated with HPD use exceeds that observed under simulated NIHL listening conditions through low-pass filtering.
